# Transcatheter aortic valve replacement alters ascending aortic blood flow and wall shear stress patterns: A 4D flow MRI comparison with age-matched, elderly controls

**DOI:** 10.1007/s00330-018-5672-z

**Published:** 2018-08-21

**Authors:** E. S. Farag, J. Vendrik, P. van Ooij, Q. L. Poortvliet, F. van Kesteren, L. W. Wollersheim, A. Kaya, A. H. G. Driessen, J. J. Piek, K. T. Koch, J. Baan, R. N. Planken, J. Kluin, A. J. Nederveen, B. A. J. M. de Mol

**Affiliations:** 1 Departments of Cardiology and Cardiothoracic Surgery, Heart Centre, Academic Medical Centre, University of Amsterdam, P.O. Box 22660, 1100 DD Amsterdam, The Netherlands; 2 Department of Radiology and Nuclear Medicine, Academic Medical Centre, Amsterdam, The Netherlands

**Keywords:** Magnetic resonance imaging, Aortic valve, Transcatheter aortic valve replacement, Cardiac surgical procedures, Thoracic aorta

## Abstract

**Background:**

With the implementation of transcatheter aortic valve replacement (TAVR) in lower-risk patients, evaluation of blood flow characteristics and the effect of TAVR on aortic dilatation becomes of considerable interest. We employed 4D flow MRI in the ascending aorta of patients after TAVR to assess wall shear stress (WSS) and compare blood flow patterns with surgical aortic valve replacement (SAVR) and age- and gender-matched controls.

**Methods:**

Fourteen post-TAVR patients and ten age- and gender-matched controls underwent kt-PCA accelerated 4D flow MRI of the thoracic aorta at 3.0 Tesla. Velocity and wall shear stress was compared between the two groups. In addition, aortic flow eccentricity and displacement was assessed and compared between TAVR patients, controls and 14 SAVR patients recruited as part of an earlier study.

**Results:**

Compared to controls, abnormally elevated WSS was present in 30±10% of the ascending aortic wall in TAVR patients. Increased WSS was present along the posterior mid-ascending aorta and the anterior distal-ascending aorta in all TAVR patients. TAVR results in eccentric and displaced flow in the mid- and distal-ascending aorta, whereas blood flow displacement in SAVR patients occurs only in the distal-ascending aorta.

**Conclusion:**

This study shows that TAVR results in increased blood flow velocity and WSS in the ascending aorta compared to age- and gender-matched elderly controls. This finding warrants longitudinal assessment of aortic dilatation after TAVR in the era of potential TAVR in lower-risk patients. Additionally, TAVR results in altered blood flow eccentricity and displacement in the mid- and distal-ascending aorta, whereas SAVR only results in altered blood flow eccentricity and displacement in the distal-ascending aorta.

**Key Points:**

*• TAVR results in increased blood flow velocity and WSS in the ascending aorta.*

*• Longitudinal assessment of aortic dilatation after TAVR is warranted in the era of potential TAVR in lower-risk patients.*

*• Both TAVR and SAVR result in altered blood flow patterns in the ascending aorta when compared to age-matched controls.*

## Introduction

In the last decade, transcatheter aortic valve implantation (TAVR) has emerged as a solid alternative for surgical aortic valve replacement (SAVR) in high- and intermediate-operative risk patients with symptomatic aortic valve stenosis (AS) [1–3]. Although clinical results after TAVR in these patients show comparable short-term results to SAVR, long-term outcomes are scarce. As we move towards the application of TAVR in lower risk, and thus probably younger and healthier, patients, post-procedural survival will increase. Therefore, any evidence on characteristics that may influence long-term outcomes, such as valve durability and aortic dilatation, is warranted.

Four-dimensional (4D) flow magnetic resonance imaging (MRI) is a novel imaging technique capable of assessing aortic blood flow in three directions as a function of time, allowing for quantification of aortic haemodynamics [4]. Various advanced parameters can be derived from 4D flow MRI-acquired velocity data that may provide novel insight into aortic haemodynamics after TAVR, such as wall shear stress (WSS), flow eccentricity and flow displacement [5–7]. A recent histological study has shown that abnormal WSS results in increased deregulation of the aortic extracellular matrix and degeneration of elastic fibres, which may result in progressive aortic dilatation [8]. Furthermore, in a study among bicuspid aortic valve disease patients, flow eccentricity has been correlated to progressive ascending aortic dilatation [9, 10]. Previous studies have reported alterations in aortic WSS distribution and flow eccentricity after both TAVR and SAVR [11]. However, no studies have been conducted comparing TAVR with age- and gender-matched controls, despite described age-related changes in aortic blood flow haemodynamics among healthy individuals [11–13].

The aim of this study was to employ 4D-flow MRI for the assessment of blood flow and WSS in the ascending aorta in patients 1 year after TAVR and compare these parameters to age- and gender-matched controls with no history of cardiovascular disease. We hypothesised that altered blood flow patterns and WSS are present after TAVR, when compared to age- and gender- matched controls. Additionally, we compared blood flow displacement and eccentricity patterns between TAVR patients, controls and SAVR patients.

## Methods

### Study population

Fourteen patients who underwent transfemoral TAVR with the SAPIEN 3 valve (Edwards Lifesciences) in the previous 18 months were included in this prospective cross-sectional study. In addition to standard MRI exclusion criteria, patients with known persistent atrial fibrillation or a history of multiple heart valve replacements were excluded. Ten age- and gender-matched individuals with no history of aortic and/or cardiovascular and/or valvular disease were included in this study. Fourteen patients in the SAVR group were treated with the Mitroflow stented bioprosthesis (LivaNova PLC, London, UK) and underwent aortic 4D flow MRI as part of a prior study conducted and published by van Kesteren et al [14]. All patients underwent aortic valve replacement (either TAVR or SAVR) due to symptomatic aortic valve stenosis.

The institutional review board approved this study and all subjects signed informed consent.

### Magnetic resonance imaging

The TAVR patients and controls underwent cardiac and respiratory-gated sagittal 4D flow MRI of the thoracic aorta at 3.0 Tesla (Philips). Standard transmit and receive cardiac coils were used for 4D flow measurements. 4D flow MRI sequence parameters were as follows: spatiotemporal resolution: 2.5 × 2.5 × 2.5 mm^3^, temporal resolution: ± 40ms (24 timeframes); TE/TR/FA = 2.1 ms/3.4 ms/8°; VENC: 150–250 cm/s; k-t PCA acceleration factor: 8. Two-al (2D) phase-contrast MRI scout measurements at the level of the sinotubular junction were conducted to estimate the optimal velocity encoding to minimise velocity aliasing. SAVR patients were included as part of a previously published study and underwent 4D flow MRI at 1.5 Tesla with scan parameters as earlier described [14].

### Data analysis – velocity and WSS

The ascending aorta was defined as the aortic segment between the aortic valve and the origin of the brachiocephalic trunk in healthy controls. In TAVR patients and controls, the ascending aorta was defined as the segment between the first circumferential area of the ascending aorta not susceptible to metal-induced artefacts and the origin of the brachiocephalic trunk. The ascending aorta was segmented and corrected for eddy currents, Maxwell terms and velocity aliasing using in-house software programmed in MatLab (MathWorks, Natick, MA, USA) [14, 15]. Mean and maximum blood flow velocity and WSS were calculated at the peak systolic time frame using previously published algorithms [16]. Due to the difference in data acquisition techniques between SAVR patients and TAVR/control group, no WSS comparison was conducted between SAVR patients and the other groups.

Cohort-averaged velocity and WSS three-dimensional (3D) ‘heat maps’ were created from the control group data, delineating elevated velocity and WSS in the aorta of TAVR patients [17, 18]. A ‘shared’ geometry of the control group was created and each aorta was co-registered to this shared geometry, followed by interpolation of systolic velocity and WSS values. After interpolation, velocity and WSS average and standard deviation (SD) values of each individual voxel were calculated. Subsequently, average and SD velocity and WSS maps of the control cohort were projected onto the aortic geometry of each individual patient. By delineating in red where the velocity or WSS values of the patient were higher than the average +1.96*SD control values, and in blue where the velocity or WSS values of the patient were lower than the average –1.96*SD control values, velocity and WSS heat maps were created. The amount of elevated WSS was expressed as the surface area with elevated WSS as a percentage of the entire surface area of the ascending aorta. Finally, the heat maps were projected on cohort-specific ‘shared’ geometries [19]. By addition of the heat maps, a 3D incidence map showing regional incidence of elevated velocity and WSS was created [18]. Aortic dimensions were calculated using a 3D surface mesh, delineating the aortic wall, which was created from the segmentation and smoothed with a Laplacian filter. Normal vectors were calculated on each point on the wall and used for: (1) 3D WSS calculation as previously described [17] and (2) 3D diameter calculation by tracking the length of the inward normal upon exiting the opposite aortic wall [18].

### Data analysis – flow eccentricity and displacement

**Fig. 1:**
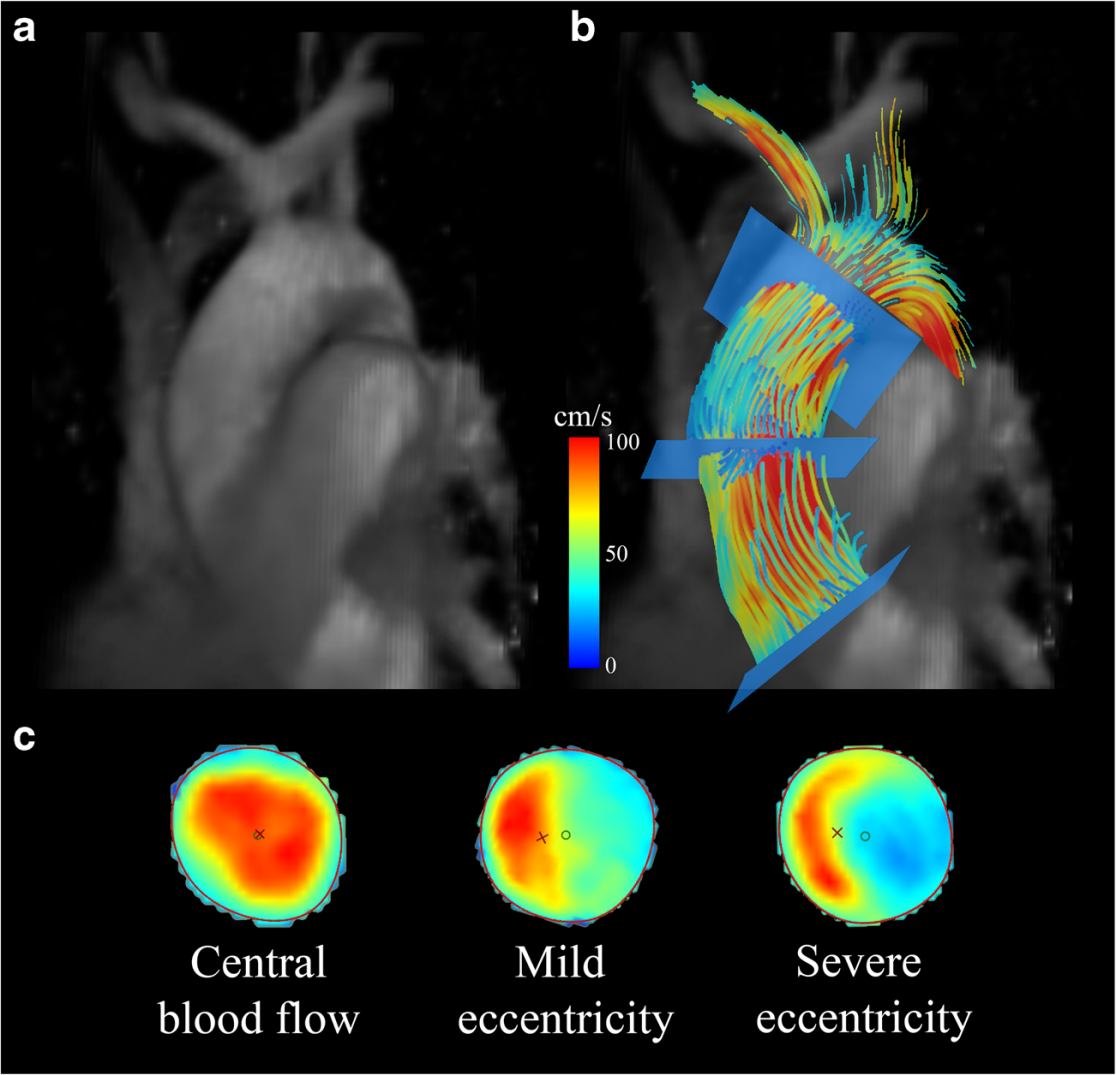
(**a**) Example of an individual control phase contrast MR angiogram in one patient. (**b**) Example of the aforementioned patient showing peak systolic pathlines of the thoracic aorta, colour-coded for velocity, with slice positioning at three locations. (**c**) Grading scale of 2D peak systolic flow maps depicting various degrees of blood flow eccentricity in the three aforementioned locations in the ascending aorta. Results of eccentricity analyses for each group are shown in Fig. 3

Commercially available software (CAAS MR 4D Flow, Pie Medical Imaging, Maastricht, The Netherlands) was used to compare blood flow eccentricity and flow displacement between groups. 2D peak systolic planes were placed at the sinotubular junction, in the mid-ascending aorta and in the distal-ascending aorta and flow displacement was calculated [9]. Blood flow displacement was defined as the distance between the centre of the lumen and the ‘center of velocity’ of the flow, normalised to the lumen diameter [9]. Blood flow eccentricity was graded semi-quantitatively by two blinded observers as previously described; central flow (high velocity flow in the majority of the vessel), mildly eccentric (high velocity flow in one- to two-thirds of the vessel lumen) and severely eccentric (one-third or less of the vessel) blood flow (Fig. 1) [13].

### Statistical analysis

Categorical variables are expressed as number (n) and percentage (%). Results were tested for Gaussian distribution using the Kolmogorov-Smirnov test. Continuous variables with a normal distribution are reported as the mean±standard deviation (SD) and continuous variables with a non-normal distribution are reported as median (interquartile range). To compare the results between the three subgroups, categorical variables were compared using Fisher’s exact test. Normally distributed continuous data were compared using the Kruskal-Wallis test. All *p*-values were two-sided and considered statistically significant if 0.05 or lower. Statistical analyses were performed using the Statistical Package for Social Sciences (SPSS, IBM analytics, Chicago, IL, USA) version 24.0

## Results

### Study participants

Participant characteristics are presented in Table 1. No significant differences in age were present between patient groups and controls (TAVR vs. control; *p* = 0.327, SAVR vs. control; *p* = 0.229), but TAVR patients were older than SAVR patients (TAVR vs. SAVR; *p* = 0.002). Except for age, baseline and demographic characteristics, cardiac risk factors, predicted surgical risk (STS-PROM and Euro SCORE-II) and echocardiographic measurements were comparable between the three groups. All groups showed similar cardiac function, with comparable end-systolic volumes and ejection fractions. However, the controls show smaller end-diastolic volumes and stroke volumes than both the TAVR and SAVR patients. Implanted prostheses sizes were comparable between the TAVR and SAVR groups (χ^2^ (2, N = 28) = 3.600, *p* = 0.165). Mean and maximum ascending aortic diameters were comparable between the three groups (Table 2). All TAVR patients underwent uncomplicated transfemoral valve implantation. Peri- and post-procedural angiograms revealed appropriate prosthesis alignment and did not show significant paravalvular leakage. Post-procedural echocardiography revealed acceptable transvalvular aortic valve gradients in all of the TAVR patients.

**Table 1:** Study participants

	TAVR (n=14)	Stented SAVR (n=14)	Controls (n=10)	*p*-value
Age (year, mean ± SD)	80.2 ± 4.7	73.9 ± 4.3	77.2 ± 4.1	0.007
Males (n (%))	5 (36%)	9 (64%)	5 (50%)	0.319
BMI (kg/m^2^, mean ± SD)	25.81 ± 4.17	25.13 ± 2.56	27.85 ± 5.02	0.154
BSA (m^2^, mean ± SD)	1.94 ± 0.2	1.89 ± 0.15	1.89 ± 0.19	0.828
Cardiovascular history and risk factors				
Hypertension (n (%))	6 (43%)	10 (71%)	3 (30%)	0.108
Hyperlipidaemia (n (%))	4 (29%)	9 (64%)	20 (20%)	0.053
Diabetes mellitus (n (%))	2 (14%)	2 (14%)	0 (0%)	0.450
Former Smoking (n (%))	4 (29%)	6 (43%)	3 (30%)	0.690
Current Smoking (n (%))	0 (0%)	3 (21%)	1 (10%)	0.181
Family history* (n (%))	4 (29%)	4 (29%)	4 (40%)	0.800
EuroSCORE-II (mean ± SD)	2.31 ± 0.97	2.05 ± 1.69	-	0.124
STS-PROM (mean ± SD)	2.715 ± 0.770	2.337 ± 1.995	-	0.015
Time between TAVR/SAVR and MRI (days, mean ± SD)	366 ± 62	361 ± 38	-	0.323
Valve size distribution			-	-
21/23/26/29 mm, n	0/9/5/0	-	-	-
21/23/25/27 mm, n	-	3/6/4/1		-
Postoperative echocardiography				
LVF class (good/mildly impaired/moderately impaired/severely impaired)	13/1/0/0	13/1/0/0	-	-
AV-peak gradient (mmHg, mean ± SD)	27.0 ± 8	21.5 ± 8	-	0.093
PVL/AR (none/trace/mild/moderate/severe)	6/6/2/0/0	12/0/2/0/0	-	-
Baseline MRI measurements				
LVEF (%, mean ± SD)	63.9 ± 7.9	65.0 ± 11.4	64.5 ± 6.4	0.845
Stroke volume (ml, mean ± SD)	89.6 ± 19.2	87.1 ± 17.2	63.6 ± 19.1	0.004
LVEDV (ml, mean ± SD)	142.4 ± 33.8	134.5 ± 18.2	99.2 ± 31.4	0.004
LVESV (ml, mean ± SD)	52.7 ± 20.5	47.3 ± 18.2	35.6 ± 14.8	0.135

*TAVR* transcatheter aortic valve replacement, *SAVR* surgical aortic valve replacement, *SD* standard deviation, *BMI* body mass index, *BSA* body surface area, *EuroSCORE* European System for Cardiac Operative Risk Evaluation, *STS-PROM* Society of Thoracic Surgery Predicted Risk Of Mortality, *LVF* left ventricular function, *AV-gradient* aortic valve gradient, *PVL* paravalvular leakage, *AR* aortic regurgitation, *MRI* magnetic resonance imaging, *LVEF* left ventricular ejection fraction, *LVEDV* left ventricular end-diastolic volume, *LVESV* left ventricle end-systolic volume

*Family history positive for cardiovascular disease in people aged < 65 years

**Table 2:** Four-dimensional flow MRI parameters

	TAVR (n=14)	Stented SAVR (n=14)	Controls (n=10)	*p*-value
Mean diameter (cm)	3.3 ± 0.3	3.3 ± 0.4	3.3 ± 0.3	0.995
Maximum diameter (cm)	4.1 ± 0.4	4.5 ± 0.6	4.2 ± 0.5	0.156
Mean WSS (Pa)	0.36 ± 0.54	-	0.24 ± 0.09	< 0.001
Peak WSS (Pa)	0.90 ± 0.25	-	0.62 ± 0.33	0.025

*MRI* magnetic resonance imaging, *TAVR* transcatheter aortic valve replacement, *SAVR* surgical aortic valve replacement, *cm* centimetre, *WSS* wall shear stress, *P*a Pascal

### Blood flow velocity and wall shear stress after TAVR

**Fig. 2:**
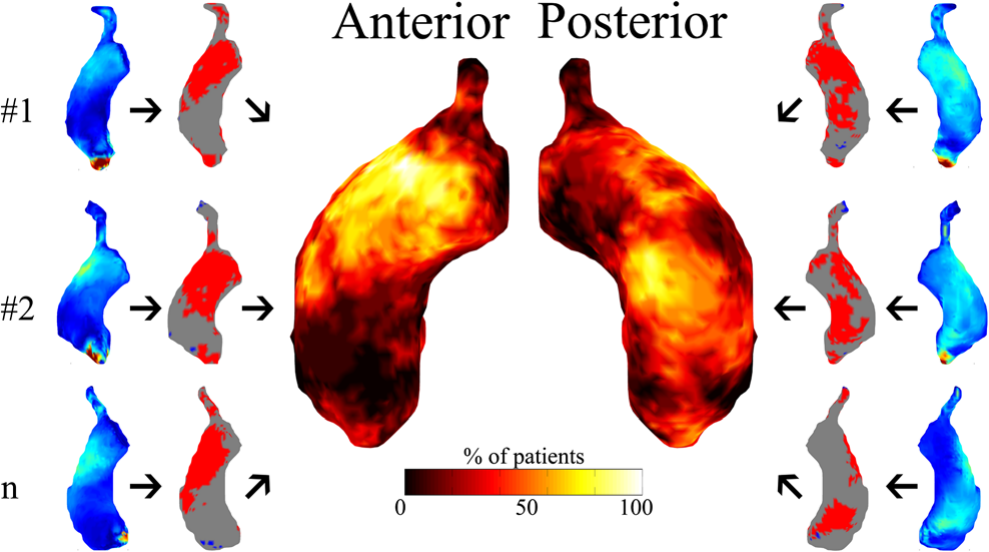
Individual patients’ (#1, #2 … #n) peak systolic wall shear stress (WSS) maps are compared with peak systolic 3D WSS atlases of controls, resulting in patient-specific WSS heat maps depicting regions with increased (red) or decreased (blue) WSS. The incidence map (centre) depicts the number of transcatheter aortic valve replacement (TAVR) patients (%) subject to increased WSS per region of the ascending aorta

Peak blood flow velocity could not be assessed in TAVR patients due to susceptibility artifacts at the level of the vena contracta caused by the metal valve stent. Mean and peak WSS were significantly higher in TAVR patients compared to the controls (Table 2). Heat maps depicting areas subject to increased velocity and wall shear stress show increased velocity and WSS in all TAVR patients. Compared to controls, abnormally elevated blood flow velocity was present in 19±8% of the ascending aortic lumen. As a result, abnormally elevated WSS was present along 30±10% of the vessel wall of the ascending aorta. Abnormally increased WSS was found in all TAVR patients on the posterior mid-ascending aorta and the anterior distal-ascending aorta, as depicted in Fig. 2.

### Flow eccentricity and displacement

**Fig. 3:**
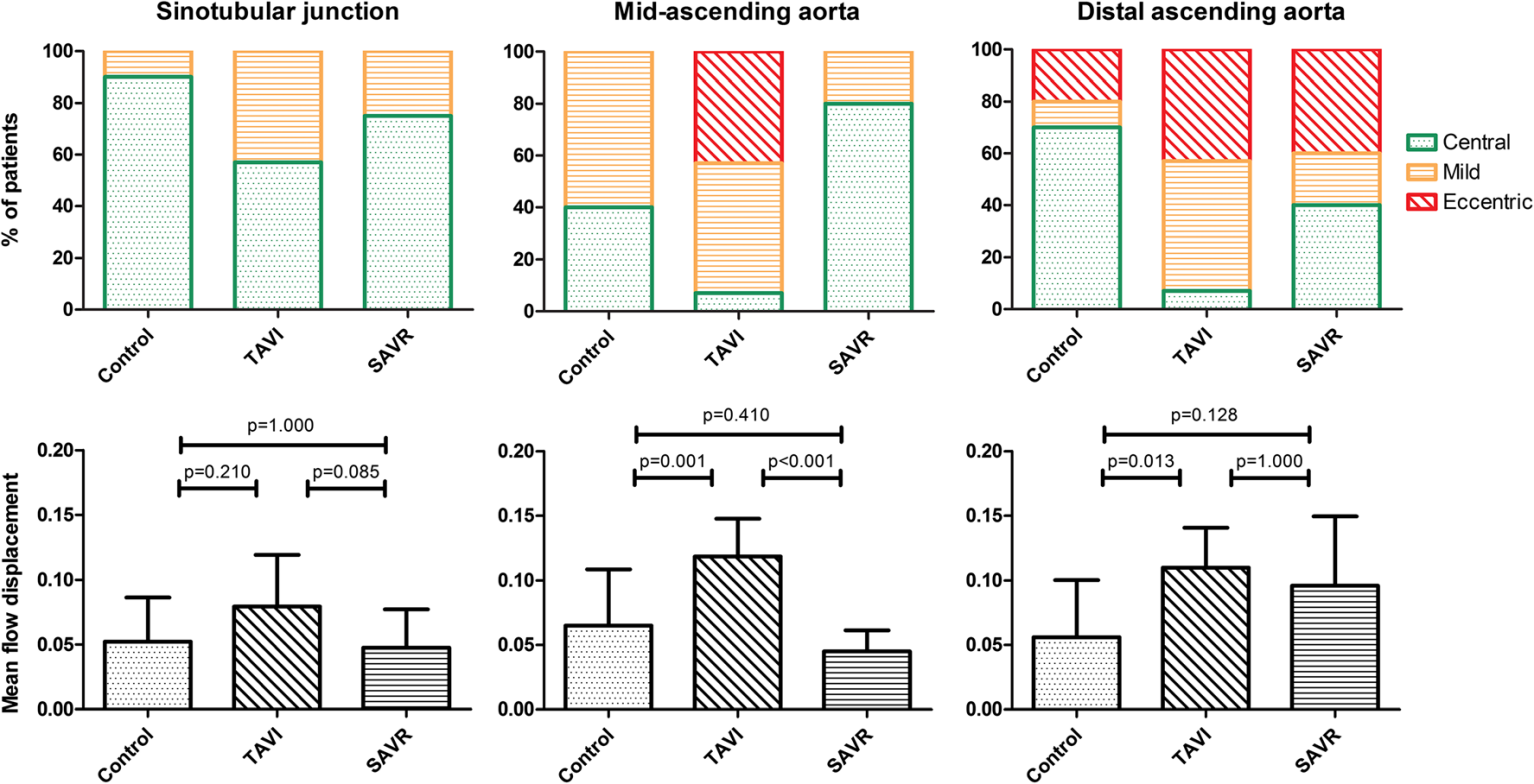
**Top**: Semi-quantitatively adjudicated degrees of blood flow eccentricity at three levels in the ascending aorta, by using the grading scale as depicted in Fig. 1C. Bottom: mean amount of blood flow displacement at three levels in the ascending aorta

Assessment of flow displacement and eccentricity was conducted successfully in all patients. All controls, except for one, demonstrated a central flow pattern at the level of the sinotubular junction, whereas only 57% and 83% showed central flow in the TAVR and SAVR patients respectively (χ^2^ (2, N = 38) = 4.025, *p* = 0.134, Fig. 3). No differences in the degree of flow displacement between groups were found at the level of the sinotubular junction.

In the mid-ascending aorta, 40% of the control patients showed central flow, compared with merely 7% in the TAVR group. Surprisingly, 84% of the SAVR patients show central flow in the mid-ascending aorta (χ^2^ (4, N = 38) = 28.041, *p* < 0.001). Significant differences were seen in the TAVR group compared with the other subgroups regarding flow displacement (Fig. 3).

In the distal-ascending aorta, severe flow eccentricity towards the outer curvature of the aorta is present in 20%, 43% and 40% of, respectively, the control, TAVR and SAVR patients (χ^2^ (4, N = 38) = 11.171, *p* = 0.025). Flow displacement values are significantly higher in TAVR patients compared to the control group (*p* = 0.013) and comparable to SAVR (*p* = 0.128).

## Discussion

In this study, we employed 4D flow MRI to study ascending aortic haemodynamics after transfemoral TAVR. This is the first *in vivo* 4D flow MRI study comparing TAVR with an age- and gender-matched elderly control group, allowing for adequate comparison of blood flow patterns and WSS. We show that: (1) TAVR resulted in increased blood flow velocity and WSS in the ascending aorta compared to age- and gender-matched controls with no history of cardiovascular disease; (2) both TAVR and SAVR resulted in altered blood flow patterns in the ascending aorta compared to age- and gender-matched controls; and (3) there were significant differences between post-procedural TAVR and SAVR blood flow eccentricity and displacement patterns.

### WSS and velocity after TAVR

In this study, we showed that TAVR resulted in increased mean and peak ascending aortic WSS when compared to controls with no history of cardiovascular disease. This finding of increased peak WSS after TAVR is in agreement with an earlier reported study among SAVR patients, when compared with younger, healthy controls showing elevated peak ascending aortic WSS [20]. Furthermore, we showed that the ascending aortic WSS was elevated in large regions of the ascending aorta and that central lumen blood flow velocity was significantly higher in all TAVR patients, when compared to our control group. This finding may be the result of two important factors. First, the balloon-expandable TAVR prosthesis is implanted inside the calcified, native aortic valve annulus. This inevitably results in a smaller effective orifice area (EOA) of the TAVR valve when compared to a healthy aortic valve. Second, slowly progressive pre-procedural aortic valve stenosis leads to left ventricular remodelling. Subsequent valve replacement (i.e. TAVR or SAVR) relieves this stenosis, lowering the needed end-diastolic pressure to overcome the aortic valve gradient, resulting in an increased stroke volume when compared to controls without any aortic valve disease [21], as we show in our baseline CMR measurements. We find that increased WSS is present in the posterior mid-ascending aorta and the anterior distal-ascending aorta in all TAVR patients (Fig. 2), which implies that post-procedural WSS alterations are inevitable. This may have important long-term clinical implications, as increased WSS induces degeneration of elastic fibres and dysregulation of the extracellular matrix of the aortic wall [8]. This may lead to progressive aortic dilatation ascending root and aorta of TAVR patients, increasing the risk of aneurysm formation or dissection. As recent clinical studies suggest, non-inferiority of transfemoral TAVR when compared to SAVR in intermediate-risk (and often younger) patients during the available short-term follow-up, accelerated aortic dilatation may have important prognostic implications, despite successful treatment of prognosis-influencing AS [22]. These findings justify scientific and clinical attention focusing on possible accelerated ascending aortic dilation after successful TAVR, reflecting favourably in long-term longitudinal follow-up studies.

### Blood flow eccentricity and displacement

In an earlier study, conducted by van Kesteren et al, blood flow patterns between stentless and stented bioprosthetic aortic valves were compared, showing blood flow patterns possibly in favour of the stentless valves, with a less obstructed profile with a significantly higher central velocity profile and lower values for outer lumen velocity and WSS [14]. However, this study was limited by the absence of an age-matched control group. By including patients with stented bioprosthetic aortic valves in our qualitative analysis, we aimed to provide a concise comparison between TAVR and conventional SAVR with an age- and gender-matched control group. TAVR resulted in eccentric and displaced flow in the mid- and distal-ascending aorta, whereas blood flow displacement and eccentricity in the SAVR predominantly occurs in the distal-ascending aorta. In a study comprising patients with BAV disease, the degree of flow displacement correlated with the aortic growth rate in these patients, proposing flow displacement as a potential risk factor for aortic dilatation [9].

Recently, Trauzeddel et al have shown that both TAVR and stented SAVR result in altered blood flow across the newly implanted valve when compared with much younger, healthy controls. In a head-to-head comparison, the stented SAVR showed significantly more distinct helices and vortices, presumably originating from the prosthesis design and smaller EOA, compared to the studied patients who received TAVR [11]. Our study also suggested different blood flow patterns, suggesting different jet directions between TAVR and SAVR patients. We hypothesise that the differences in location and degree of flow displacement and eccentricity originate from the implantation technique of the prosthetic valves. SAVR valves are implanted under direct sight, allowing for optimal angulation of the valve. This results in a blood flow direction that is similar to a native aortic valve. However, due to the increased blood flow velocity caused by the smaller EOA, flow displacement occurs in the distal-ascending aorta. In contrast, TAVR is a transcatheter technique performed with angiographic imaging only. This possibly induces increased blood flow eccentricity and displacement occurring earlier, in the mid-ascending aorta. In the distal-aorta, TAVR and SAVR show a comparable amount of flow displacement and eccentricity, although both are significantly higher than in controls. Newer TAVR-prostheses could possibly reduce the extent of increased velocity and WSS originating from the jet caused by the prosthesis itself. However, improved valve design may probably not completely annul this, as it is an inevitable consequence of the calcification of the native valve, the prosthesis design and the minimally-invasive approach.

### Limitations

As with many 4D flow studies, this study is limited by its sample size. Furthermore, as earlier mentioned, we were unable to compare WSS values and patterns between TAVR and SAVR patients due to differences in acquisition parameters. For example, it is known that WSS estimations are highly dependent on spatial resolution [16, 23]. Since the voxel volume of the SAVR datasets is 7.5 mm^3^ (=1.5 mm × 1.5 mm × 1.5 mm), compared to 15.6 mm^3^ (= 2.5 mm × 2.5 mm × 2.5 mm) of the control and TAVR datasets, differences in WSS between SAVR and TAVR will likely be caused by differences in spatial resolution. Furthermore, differences in scanner hardware (gradient systems, coils), acquisition parameters (TE, TR) and data processing (background phase offset correction) prohibit further quantitative comparison for velocity and WSS. This prevented us from analysing velocity and WSS quantitatively between the three groups. 4D-flow MRI data was not available prior to TAVR or surgery, which prevented us from analysing actual alteration in WSS and flow patterns. However, our findings of increased blood flow velocity and WSS in the ascending aorta justify scientific and clinical attention focusing on possible accelerated ascending aortic dilation after successful TAVR. Finally, cardiac baseline parameters (left-ventricular end-diastolic volume and stroke volume) were significantly higher in TAVR and SAVR patients compared to controls.

## Conclusion

This study showed that TAVR results in increased blood flow velocity and WSS in the ascending aorta compared to age- and gender-matched elderly controls. As younger patients may undergo TAVR in the coming decades, the clinical implications of our findings of altered blood flow and WSS patterns requires scientific and clinical attention. Long-term longitudinal follow-up studies, imaging the ascending aorta after TAVR and assessing aortic dilatation are warranted. Additionally, TAVR results in altered blood flow eccentricity and displacement in the mid- and distal-ascending aorta, whereas SAVR only results in altered blood flow eccentricity and displacement in the distal-ascending aorta.

## Abbreviations

3D: Three-dimensional
4D: Four-dimensional
AS: Aortic valve stenosis
EOA: Effective orifice area
MRI: Magnetic resonance imaging
SAVR: Surgical aortic valve replacement
SD: Standard deviation
TAVR: Transcatheter aortic valve replacement
